# The Dependence of Isothermal ω Precipitation on the Quenching Rate in a Metastable β-Ti Alloy

**DOI:** 10.1038/srep14632

**Published:** 2015-10-09

**Authors:** Jing Chen, Wenlong Xiao, Matthew Simon Dargusch, Chaoli Ma

**Affiliations:** 1 Key Laboratory of Aerospace Advanced Materials and Performance of Ministry of Education, School of Materials Science and Engineering, Beihang University, Beijing 100191, China; 2 The University of Queensland, Centre for Advanced Materials Processing and Manufacturing (AMPAM), Brisbane QLD 4072, Australia

## Abstract

The precipitation behavior of the α strengthening phase in metastable β-Ti alloys is highly dependent on heat treatment parameters such as quenching rate, heating rate and ageing temperature. In this paper we have investigated the influence of quenching rate on the formation of isothermal ω precipitates that have been regarded as potent nucleant sites for α precipitation. The results show that the β-solutionized alloy contains a β matrix with a layer structured morphology. Regular atomic movement of the (002)_β_ plane along the <002> direction was observed in the alloy. The increase in quenching rate refines the thickness of layers, subsequently influencing the nucleation and growth of isothermal ω precipitates after ageing treatment. The high quenching rate promotes the occurrence of ω precipitation, broadens the stage of ω precipitation and increases the number density of ω precipitates. Since the isothermal ω phase provides a heterogeneous nucleation site for α precipitates, it is inferred that the quenching rate may indirectly influence the mechanical properties of metastable β-Ti alloy.

Metastable β-Ti alloys are one of the most important classes of Ti alloys for aerospace and other high performance structural applications because of their high strength to weight ratios and excellent corrosion resistance^1^,^2^. Their properties including strength and toughness are influenced by the morphology, size, volume fraction and distribution of α precipitates, which are critically determined by heat treatment parameters such as heating rate, ageing temperature and time, etc.^3^,^4^,^5^,^6^. It has shown that the slow heating rate to reach the isothermal ageing temperature increases the number of potent nucleant ω phase for α phase precipitation, leading to the formation of finer and more uniform distributions of α precipitates^7^,^8^,^9^,^10^. The presence of such fine and uniform α precipitates will have a notable influence on the mechanical properties of β-Ti alloys^11^,^12^,^13^. Because of the metastable microstructure and low thermal conductivity of Ti alloys, in industry practice, it is imaginable that the heat treatment history such as cooling rate and heating rate, may be much different depending on locations from the external areas to the core of large Ti components. Such variation will finally develop different microstructures and inevitably result in different mechanical properties at different locations. Therefore, attempts to understand the evolution of microstructure of the metastable β-Ti alloy under different heat histories has gained great scientific interest. In this paper, we investigate the influence of quenching rate on the formation of isothermal ω precipitates in a newly developed metastable β-Ti (Ti-6Cr-5Mo-5V-4Al) alloy. We show that the quenching rate has an obvious effect on the formation of the isothermal ω phase, which will potentially influence the nucleation of α strengthening precipitates and indirectly influence the final mechanical properties of the metastable β-Ti alloy.

## Results and Discussion

**Figure 1:**
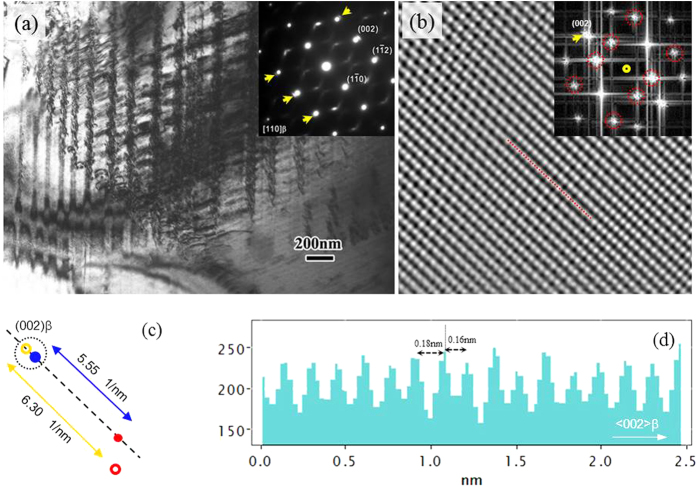
(**a**) Bright-field TEM image and SAED pattern of AC sample; (**b**) High resolution TEM and FFT images showing the existence of extra diffraction spot near (002)_β_; (**c**) The schematic diagram showing the distance between the transmission spot and the (002)_β_ diffraction spot and the extra diffraction spot; (**d**) The atomic displacement was calculated using an intensity profile along the <002>_β_ direction.

The β transus (the temperature at which the α + β → β phase transformation is complete) of the studied alloy is ~760 °C, and the microstructure is expected to be fully be transformed into the β phase at 830 °C, however, due to the metastable nature of the alloy, a phase transformation may occur during cooling to room temperature. Figure 1(a) shows the TEM bright-field image taken from the AC sample. It can be seen that the AC alloy exhibits a layered β matrix with the thickness of layers varying from ~100–200 nm. The reflections at the 1/3 and 2/3 (211)_β_ position can not be observed in the corresponding selected area diffraction (SAED) pattern, indicating that the ω phase was not formed under the AC condition^14^. Solute partitioning was observed using nano-beam EDS analysis, in which the region with lighter contrast is depleted of β-stabilizer. The solute partition is linked to the phase separation that occurs during cooling, which has been observed in other β alloys^15^,^16^. Because of the metastable phase stability, the phase equilibrium established at the solid solution temperature will be disrupted during cooling, and the elements diffuse like spinodal decomposition via a designated crystallographic direction to reduce the free energy of the whole system^15^,^17^.

Under the AC condition, the phase separation seems to be incomplete because of the relatively high phase stability and the limited time available for diffusion during cooling. Extensive reciprocal lattice streaking (RLS) and extra weak diffraction spots marked with arrows near the {002} and {112} positions along the <002> direction can be observed in the SAED pattern (Fig. 1a). Such extra diffraction spots have been found in other metastable β-Ti alloys^18^. Figure 1(b) shows the high resolution TEM image of the AC alloy. Extra diffraction spots near (002)_β_ along the <002>_β_ direction (marked by arrow) can be confirmed in the Fast Fourier Transformation (FFT) image. As illustrated in Fig. 1(c), the distances between the (002)_β_ diffraction spot, the extra diffraction spot and the transmission spot are measured to be 5.55 1/n and 6.30 1/n, respectively. The atomic distance was calculated using an intensity profile along the <002>_β_ direction. Figure 1(d) shows the intensity profile along the dash line marked in Figure 1(b). It is indicated that two different interatomic distances (0.16 nm and 0.18 nm) periodically appear. These distances are identical to the reciprocal of the distances shown in Fig. 1(c) respectively. Hence, the sets of the extra diffraction spots (Fig. 1(a)) are considered to be the reflection of regular atomic movements. The bracket-like RLS (Fig. 1(a)) is reported to be a congregation of diffuse scattering and partially collapsed ω^19^,^20^,^21^. At sufficiently low temperatures, the RLS will be transformed into the ω phase by a displacive type reaction^20^. The spots marked by a dash circle in the FFT image (Fig. 1b) are part of the congregation (RLS) mentioned above and also caused by an incomplete lattice shift.

**Figure 2:**
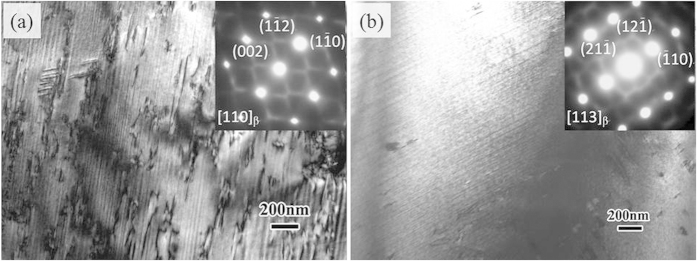
TEM image and corresponding SEAD pattern of (**a**) WQ and (**b**) LNQ samples showing that the layered structure was refined by increase of quenching rate.

Figure 2 shows the TEM bright field images of the WQ and LNQ samples. Both the WQ and the LNQ samples show regular layered structure of the β matrix, which is similar to the AC sample. The increase of quenching rate refines the layers of the β matrix. The thickness of layers in the WQ sample and the LNQ sample is ~30 nm and ~20 nm, respectively. The refinement is attributed to the increase in quenching rate which shortens the time for elemental diffusion during phase separation, thus decreasing the diffusion distance of alloying elements. In other words, the high quenching rate leads to lower degree of solute partition in the alloy. As shown in Figure 2, there is no evidence for ω precipitates observed in the TEM images and SAED patterns of the WQ and LNQ samples, while the intensity of the RLS increased with increasing quenching rate.

**Figure 3:**
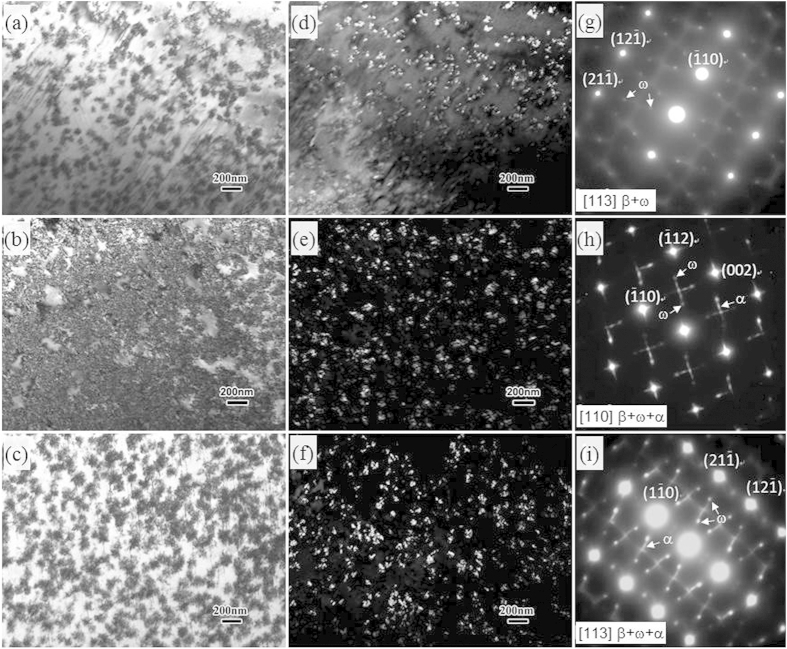
Bright-field TEM image of (**a**) AC, (**b**) WQ and (**c**) LNQ alloys after ageing treatment showing the formation of precipitates; Dark-field TEM image of the aged (**d**) AC, (**e**) WQ and (**f**) LNQ alloys taken from the reflection of ω phase; Corresponding SAED pattern of (**g**) AC, (**h**) WQ and (**i**) LNQ alloys.

The solution treated samples with different quenching rates were aged at 300 °C for 1440 min. Figure 3 shows the TEM images taken from the aged samples. The layered structure has almost disappeared, instead, a large number of precipitates were formed in all the aged samples, as shown from the bright-field TEM images in Figure 3(a–c). Although aged at the same parameters, the quenching rate apparently influences the formation of the ω precipitates. Figure 3(g–i) shows the SAED patterns taken from the alloys with different quenching rates. Reflections at 1/3 and 2/3 (211)_β_ positions that belong to ω precipitates are visible in all the alloys, which is consistent with the previous report^14^. However, except for ω precipitates, the precipitation of α phase occurs in the WQ and LNQ samples, as indicated in Figure 3(h,i). Figure 3(d–f) shows the TEM dark field images obtained using reflections from the position of 2/3 (112)_β_ that is considered as the reflections from the ω phase. The number density of the ω precipitates is increased when increasing the quenching rate. Since the ω precipitates normally nucleate and grow from the β-stabilizer depleted region^15^, the increase in number density of ω precipitates can be attributed to the refinement of the layered structure by increasing the quenching rate.

**Figure 4:**
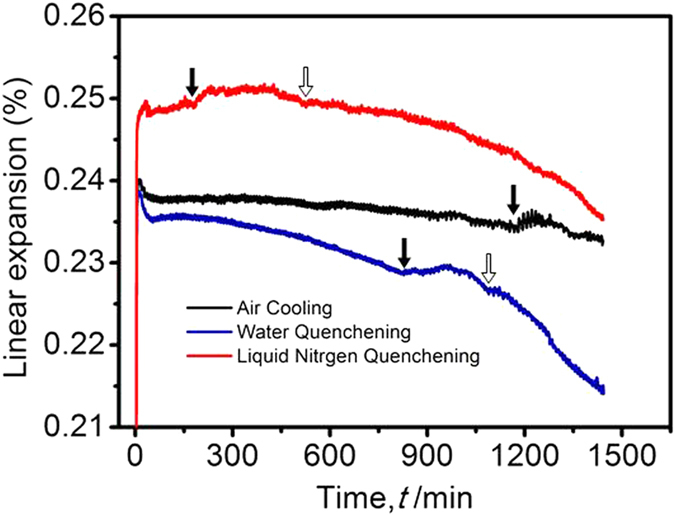
The linear expansion vs. ageing time curves of alloys cooled by different quenching rates; The solid arrows showing the occurrence of ω precipitation, and the open arrows showing the emergence of α precipitation.

A thermal mechanical analyzer (TMA) has been used to study more details on the influence of quenching rate on the ω precipitation. Figure 4 shows the linear thermal expansion vs. time curves under the isothermal process at 300 °C. The sudden change in linear expansion is caused by phase transformation. The linear expansion of all the samples reaches a sharp peak after heating to the ageing temperature, and then slightly decreases within several minutes due to thermal balance in the whole sample. However, with increasing ageing time, the linear expansion of the AC and LNQ samples becomes almost stable, while that of the WQ sample gradually decreases before sudden change. The decrease in linear expansion of the WQ sample may be related to dislocation recovery. As shown in Fig. 2(a), a relatively high density of dislocations are present in the WQ sample, but it is still unclear why a high density of dislocations is solely obtained under an intermediate cooling rate (WQ).

A sudden change in linear expansion occurs in all the alloys with increasing ageing time, indicating that the ω precipitates start to nucleate and grow. It is found that the time taken for ω precipitation to occur is highly dependent on the quenching rate. As is shown by the solid arrows in Figure 4, the ω precipitates begin to be formed at 1176 min, 820 min and 176 min for the AC, WQ and LNQ samples, respectively. The nucleation of ω precipitates originates from lattice disturbance. The shorter time taken for ω precipitation to occur with the increasing quenching rate can be attributed to the increase of RLS, as shown in Figure 2.

As shown in Figure 4, only one peak for phase transformation is visible within the range of testing time (1440 min) in the AC sample, however, an extra peak (marked by an open arrow) can be observed after the ω precipitation is detected in the WQ and LNQ alloys. The emergence of the extra peak is regarded as the precipitation of α phase, which is consistent with the fact that there is evidence for the diffraction of α phase observed in the WQ and LNQ alloys, as shown in Figure 3(h,i). The peak width represents the stage of phase transformation. The peak width of ω precipitation in the AC, WQ and LNQ samples has been measured to be ~166 min, ~274 min and ~346 min, respectively. Therefore, it is shown that an increase in quenching rate prolongs the stage of ω precipitation. This phenomenon, can be attributed to the growth of isothermal ω precipitates, controlled by elemental diffusion, whereas the high quenching rate results in a low degree of solute partition in the solid solution treated alloy, thus leading to long term controlled diffusion mechanisms for the growth of ω precipitates.

In conclusion, the layer structured morphology of the β matrix was observed in the β-solutionized metastable β-Ti alloy. The increase in quenching rate shortens the time to elemental diffusion, refining the thickness of layers. More interestingly, it is found that the formation of isothermal ω precipitates is highly dependent on the quenching rate. The high quenching rate shortens the occurrence time for ω precipitation, broadens the stage of ω precipitation and increases the number density of ω precipitates. Since the ω precipitates can act as potent α lath nucleation sites, it is inferred that the quenching rate may indirectly influence the mechanical properties of metastable β-Ti alloy.

## Methods

Cylinders of Ti-6Cr-5Mo-5V-4Al alloy with ϕ10 mm × 10 mm in size were solid solution treated at 830 °C for 30 min, followed by air cooling (AC), water quenching (WQ) and liquid nitrogen quenching (LNQ), respectively. The solution treated samples with different quenching rates were subsequently aged at 300 °C for 1440 min. The microstructures of both solution treated samples and aged samples were observed by transmission electron microscopy (TEM) coupled with energy dispersive spectrometer (EDS). Specimens for TEM observations were mechanically polished and then milled using twin-jet electrolytic polishing. Linear thermal expansion was used to monitor the ageing response of solution treated samples. Cylindrical samples with dimensions ϕ3 mm × 6 mm in size were cut from the solution treated samples by EDM wire cutting. The samples were mechanically ground by abrasive paper in order to remove the oxides from the surface and then used for linear thermal expansion tests. The linear thermal expansion tests were conducted usinga TA Q400 thermo-mechanical analyzer (TMA). The solution treated samples were heated to 300 °C at a heating rate of ~200 °C/min and then held at 300 °C for 1440 min.

## Additional Information

**How to cite this article**: Chen, J. *et al*. The Dependence of Isothermal ω Precipitation on the Quenching Rate in a Metastable β-Ti Alloy. *Sci. Rep*. **5**, 14632; doi: 10.1038/srep14632 (2015).
